# Transplanted Moss *Hylocomium splendens* as a Bioaccumulator of Trace Elements from Different Categories of Sampling Sites in the Upper Silesia Area (SW Poland): Bulk and Dry Deposition Impact

**DOI:** 10.1007/s00128-018-2429-y

**Published:** 2018-09-11

**Authors:** Grzegorz Kosior, Aleksandra Samecka-Cymerman, Anna Brudzińska-Kosior

**Affiliations:** Department of Ecology, Biogeochemistry and Environmental Protection, Wrocław University, ul. Kanonia 6/8, 50-328 Wrocław, Poland

**Keywords:** *Hylocomium splendens*, Biomonitoring, Trace elements, Categorized study sites, Moss transplants, Bulk and dry deposition

## Abstract

Concentrations of Cd, Co, Cr, Cu, Ni, K, Fe, Mn, Pb, V and Zn in transplants of *Hylocomium splendens* (Hedw.) Schimp. were compared with bulk deposition and dust samples from three different categories of sites: industrial, residential and their surroundings and rural (15 in total). Mosses were transplanted for 90 days to severely polluted areas of Upper Silesia, and samples of precipitation and dust were collected during the same period. Most of the significant correlations between element concentrations in mosses and bulk deposition (Cd, Ni, Pb and Zn) were found for industrial sites. In this study dry deposition carried higher element concentrations than bulk deposition, which may result in the higher passive capture of particulate matter by mosses.

In Poland as well as in other European countries, air quality measurement is based on physical and chemical techniques. However, there are technical difficulties in the measurement and analysis of trace elements in the air; moreover such studies are very expensive (Ares et al. 2012). An alternative method for measuring integral trace element deposition is the use of terrestrial mosses as biomonitors. A biomonitor is an organism (or part of an organism or a community of organisms) which contains information on the quantitative aspects of the environmental quality (Markert et al. 2003). Biomonitoring is a continuous observation of an area with the help of bioindicators, e.g., by repeated measurement of their responses in a manner that reveals changes over space and time (by measuring the xenobiotics taken up, for example). Analysis of plants has many advantages in comparison with the traditional analysis of abiotic matrices (water, soil). It is possible to obtain accurate information about the contamination degree of the studied area or site. The moss technique has several advantages over precipitation analysis; sampling is easy and no expensive equipment is needed, and high element concentrations in mosses lead to simpler analysis and fewer contamination problems (Berg et al. 1995). Mosses have no root system, and they take up essential elements directly from the air (Steinnes et al. 1994). The technique of analyzing the contents of contaminants in mosses is known as passive biomonitoring (using moss that grows naturally in a particular area). Moss transplants (by transplanting moss from other locations) are used as active biomonitors. Transplants are often used because of the absence of native mosses (i.e. industrial and urban areas). For active biomonitoring, moss samples are collected from relatively unpolluted habitats exposed in a different environment. The use of moss transplants resolves various problems associated with the use of native moss. In addition, it reduces the high degree of variability in the uptake of contaminants by native moss within the same sampling site (Fernández et al. 2002). Transplants can be used more conveniently for interpreting temporal variability in the results. When moss transplants are used, initial concentration is known with a well-defined exposure time. In this investigation we used samples of transplanted mosses *H. splendens* (Hedw.) Schimp. This species is a bryophyte widely used for biomonitoring (Steinnes et al. 1994). A few comparisons are available between mosses collected from various habitats with trace element contamination as a basic criterion. The main objective of the present study was to test the ability of *H. splendens* transplants to bioaccumulate atmospheric trace elements from representatives of three different habitat categories under gradient pollution: industrial, residential and their surroundings and rural groups of sites. The goal of this study was also to examine how the concentration of trace elements in moss transplanted to contaminated sites was related to local bulk and dry deposition. We also wanted to check what concentrations can be expected for trace elements in terrestrial moss samples and what was their background/contamination ratio in the three categories of sites. The hypotheses in this study are: (1) significant differences occur in the concentration of the elements studied in the samples of moss transplants, precipitation and dust collected from the three categories of areas (industrial, urban and rural); (2) the concentration of the analyzed elements in moss depends to a different degree on the concentration of elements in the precipitation and dust samples collected from the same research stands.

## Materials and Methods

**Fig. 1:**
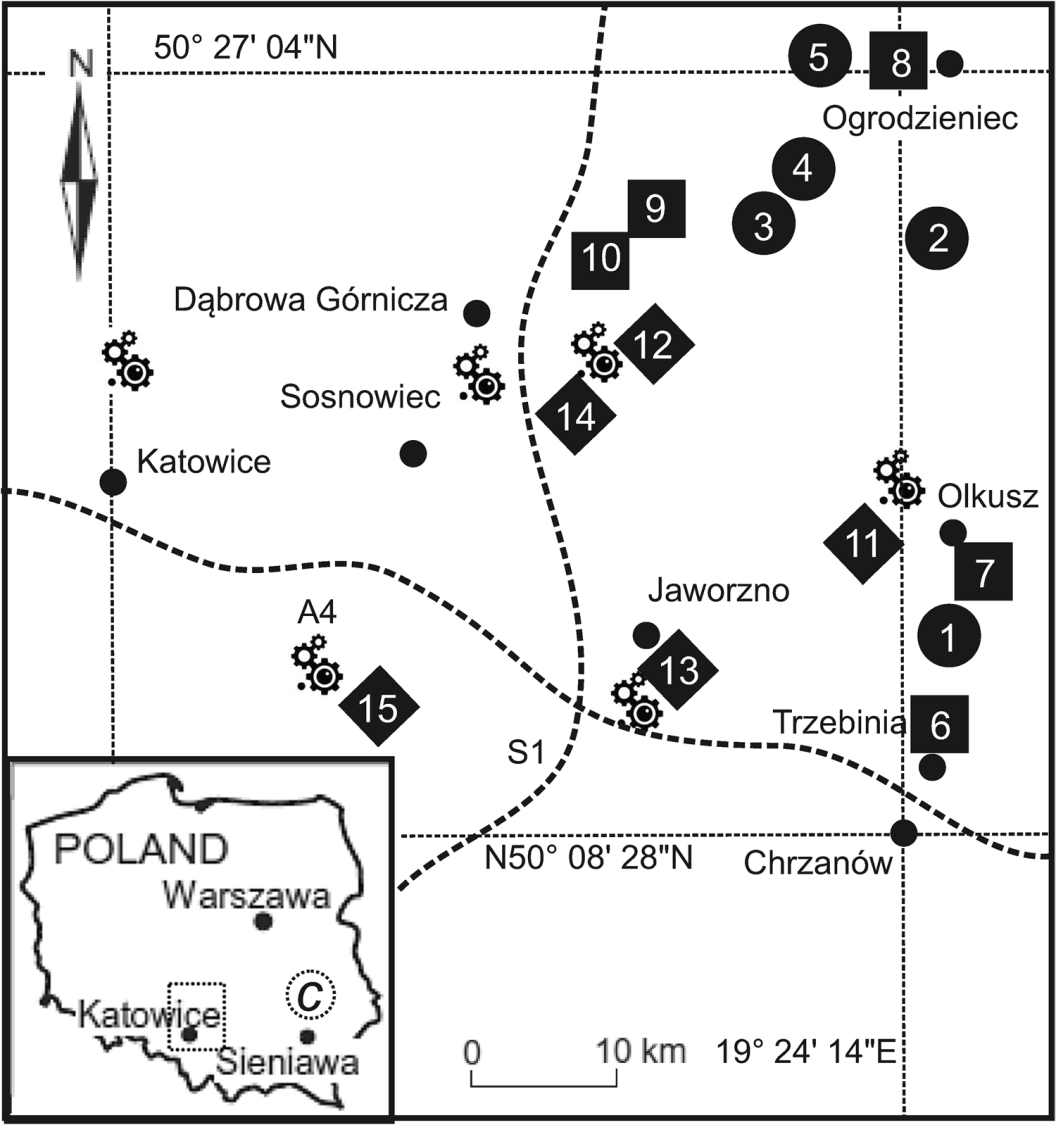
Location of the study area. Symbols: filled square – rural, filled circle – urban, filled diamond – industrial, C – control

The area of our study was Upper Silesia, the most urbanized region of Poland and one of the largest urban and industrial areas of Central Europe. Long-term exploitation of natural resources in this region together with industrialization and urbanization has caused its physical and chemical degradation, which has in turn resulted in large geochemical anomalies (Magiera et al. 2007). Samples of moss *H. splendens* were collected together with the base from 50 m × 50 m squares in a relatively clean site near Roztoczański National Park (50°19′32″N, 22°73′96″E). Subsequently, moss samples were transplanted to three different categories of sites in five replicates: rural (no. 1–5), urban and their surroundings (no. 6–10) and industrial (no. 11–15) in the Upper Silesia region (Fig. 1). The study area consisted of three groups of sites in which strong pollution gradients occurred (Kosior et al. 2010). The use of moss transplants placed directly on the ground (self-irrigated) removes certain environmental stressors (mainly hydric stress). The mosses were placed on the ground at least 5 m away from the canopy of trees so as not to be directly exposed to throughfall precipitation. The design of moss sample sites was based on the protocol adopted within UNECE ICP Vegetation, (2003). At the studied sites, five sub-samples of moss transplants were placed within a 30 × 30 m^2^ area. Transplants were exposed for 3 months (90 days). Samples were collected for analysis after 45 and 90 days. To establish a direct relationship with atmospheric deposition rain water samples were collected to determine trace element concentration. Water samples were collected in the open area into polyethylene bottles (2 L) placed 1.5 m above the ground, with funnels (10 cm diameter) on the top with nylon mesh. The bottles contained thymol as a biocide to eliminate microorganisms and five bottles were placed at each sampling site. Water samples were collected from sites immediately after precipitation and stored in a refrigerator (Hou et al. 2005). After collecting, water samples were immediately filtered to exclude particles greater than 0.45 micrometers with a Whatman filter (qualitative cellulose type 2). Pooled water samples were analyzed for elements after 45 and 90 days from the start of the experiment. In this study the method for estimating dust deposition with the help of plates coated with adhesive white petrolatum was applied (Olszowski et al. 2012). The technique enables measurement of the dust deposition amount and further particle composition analysis and involves a low cost of measurements. Measuring plates made of glass of 80 mm diameter were used. The glass plates were coated with aluminium foil in a manner to ensure a smooth surface, and then a smooth coat of the adhesive was applied. After application of white petrolatum, the plates were dried for 8 min in a dryer at 42°C to dissolve the adhesive and obtain an evenly smooth surface. Such prepared plates were weighed with 0.2 mg accuracy and five plates were placed at every sampling site. In the second stage, chemical composition of the deposited dust was determined. After completion of weighing, the plates were put in a polytetrafluoroethylene container and washed with hexane to extract particles. The solution was then filtered through an ash-free Whatman filter (qualitative cellulose type 2). The filter was dried and burnt in a crucible. The residues remaining after the burning and determination of the elements were subjected to mineralization (Olszowski et al. 2012).

The total content of the elements was determined in the transplanted *H. splendens*, rain water and dry dust. Initially the content of the elements was determined in mosses for transplantation to estimate the background values from their natural growth. After 45 and 90 days of exposure the content of elements was analyzed to determine the contamination of the selected 15 sites. To conduct the analysis of elements the moss and dust samples were digested with 3 mL of nitric acid (ultra pure, 65%) and 2 mL of perchloric acid (ultra pure, 70%) in a CEM Mars 5 microwave oven. The plant digests were analyzed for Fe, K, Mg, Mn, Zn, using Flame Atomic Absorption Spectroscopy (FAAS), Cd, Co, Cr, Cu, Ni, Pb and V using Electrothermal Atomic Absorption Spectroscopy (ETAAS) with a GF3000 Graphite Furnace, both with an AVANTA Atomic Absorption Spectrophotometer from GBC Scientific Equipment (Lajunen and Peramaki 2004). The samples of water and dust were analyzed for Cd, Co, Cr, Cu, Fe, Mn, Ni, Pb and Zn using the same methods as for mosses. The accuracy of the methods applied for the determination of the elements was checked by the analysis of Certified Reference Materials of moss M2 and M3 prepared in Finland for the European Moss Survey (Finnish Forest Research Institute). Rain water quality for the determinations was checked by simultaneous analysis of the SPS-SW1 Batch 123 reference material for the measurement of elements in surface waters (Spectrapure Standards as, Norway).

To assess element accumulation in moss transplants, relative accumulation factors (RAF) were calculated as the moss content of each element after exposure (C_exposed_) (90 days) reduced and divided by the element content before exposure (C_initial_) (0 days):

$${\text{RAF}}=\left( {{{\text{C}}_{{\text{exposed}}}}-{\text{ }}{{\text{C}}_{{\text{initial}}}}} \right)/{{\text{C}}_{{\text{initial}}}}$$

Lilliefor’s modifications of the Kolmogorov–Smirnov test were used to check the normality of the raw data, after applying Box–Cox transformations. To study the variability of the elemental concentrations within sampling sites ANOVA analysis was performed on Box–Cox transformed data to obtain normal distribution (Zar 1999). Comparisons of element concentrations in mosses, precipitation and dust samples were examined with the t-test applied on Box–Cox transformed data. Pearson correlation coefficients were calculated (Sokal and Rohlf 2003) to examine relationships between element concentrations in bulk/dry deposition and mosses after 90 days. All statistical tests were performed with Statsoft Statistica 13.

## Results and Discussion

Descriptive statistics of element concentrations in moss transplants, bulk and dust deposition are presented in Tables 1 and 2, respectively.

**Table 1:** Average (x̅), (mg kg^−1^), standard deviation (SD), relative accumulation factor (RAF) and RAF standard deviation (RAF SD) of element concentrations in *H. splendens* from control (C), rural (R – filled square, see Figs. 1, 2), urban (U – filled circle) and industrial (I – filled diamond) sites on day 90 (M 90) of the experiment (n = 60)

	x̅	SD	x̅	SD	RAF	RAF SD	x̅	SD	RAF	RAF SD	x̅	SD	RAF	RAF SD
	M C		M 90 R				M 90 U				M 90 I			
Cd	0.3	0.1	1.2	0.8	2.9	2.7	1.3	0.7	3.1	2.8	1.8	0.9	4.8	2.8
Co	0.2	0.1	0.3	0.1	0.6	0.7	0.4	0.2	0.8	2.3	0.6	0.4	2.2	0.6
Cr	1.7	0.5	2.1	0.8	0.2	0.4	2.8	1.4	0.7	0.8	2.3	0.7	0.3	0.4
Cu	8.4	1.2	9.9	2.4	0.2	0.3	10.5	2.6	0.2	0.5	13.5	3.1	0.6	0.3
Fe	557	178	1004	324	0.8	0.6	1070	524	0.9	2.8	2396	1404	3.3	1.4
K	2939	393	3420	689	0.2	0.2	3564	1512	0.2	0.5	3089	404	0.1	0.1
Mg	1048	226	1764	757	0.7	0.7	1721	675	0.6	1.9	2823	1791	1.7	0.5
Mn	582	178.2	384	202	− 0.3	0.3	539	293	− 0.1	0.4	473	355	− 0.2	0.7
Ni	1.6	0.4	1.8	0.5	0.1	0.3	2.3	0.6	0.4	1.1	3.3	1.7	1.0	0.4
Pb	5.9	2.5	34	32	4.8	5.5	26	13	3.4	3.7	39	21	5.6	2.6
V	1.8	0.3	1.6	0.4	− 0.1	0.2	2.2	0.9	0.3	0.5	2.4	0.6	0.4	0.4
Zn	61	7	194	158	2.9	2.6	138	65	1.3	2.4	258	139	3.2	1.5

**Table 2:** Average (x̅), and standard deviation (SD) of element concentrations in total precipitation samples (µg L^−1^), (bulk deposition BD, n = 60) and in dust samples (mg kg^1^), (DS, n = 60) from control (C), rural (R), urban (U) and industrial (I) sites on day 90 (90) of the experiment

	x̅	SD	x̅	SD	x̅	SD	x̅	SD		x̅	SD	x̅	SD	x̅	SD	x̅	SD
	BD C		BD90R		BD 90 U		BD 90 I			DS C		DS 90 R		DS 90 U		DS 90 I	
Cd	0.45	0.03	0.9	0.7	1	1.1	1.1	0.7	Cd	1.0	0.1	1.1	0.6	0.9	0.5	1.5	0.7
Co	0.39	0.03	0.5	0.2	0.3	0.3	0.3	0.2	Co	0.9	0.0	1.0	0.2	1.0	0.4	1.8	0.4
Cr	0.17	0.01	0.2	0.2	0.2	0.2	0.7	0.1	Cr	1.1	0.1	1.3	0.6	1.5	1.3	2.9	1.4
Cu	4.9	0.3	5	6	7	6	8	2	Cu	6.7	0.3	7.7	2.4	10.1	3.7	17	7.5
Fe	18	1	15	29	6	6	30	30	Fe	441	19	358	104	378	289	666	186
Mn	38	1	50	50	30	40	40	20	Mn	16.7	1.6	12.8	7.7	15.7	13	5.9	5.6
Ni	1.7	0.1	2	2	2	2	4	3	Ni	0.9	0.1	1.2	0.5	4.3	2.5	5.9	4.5
Pb	2	0.1	4	3	2	2	9	2	Pb	15.3	1.4	15.2	6.3	21.2	16	40	18
Zn	53	5	90	100	70	80	140	110	Zn	31	1.2	25	22	35	33	50	29

**Fig. 2:**
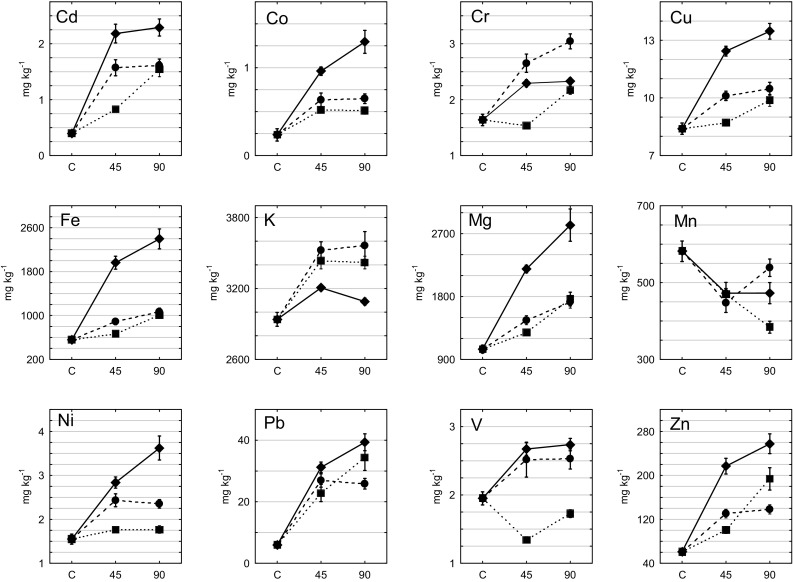
Element values (x̅) in *H. splendens* after 0 (C), 45 and 90 days of exposure in rural (filled square), urban (filled circle) and industrial (filled diamond) sites. The concentration of elements on day 0 in the transplanted *H. splendens* is represented by the value in the moss in the control site (C) from which the transplanted mosses were collected

Compared with European mosses collected from 28 countries during ‘Moss survey 2005’ (Harmens et al. 2008), mosses from this study had higher concentrations than those for European mosses for Cd, Cu, Fe, Pb, Zn for mosses collected from all sites and higher concentrations for Cr, Ni, V for mosses collected from all sites except control and rural sites. This suggests that the study sites from Upper Silesia can be placed in a group of top polluted regions in Europe with the elements mentioned above. The highest levels for most elements were determined for mosses transplanted to industrial category sites (Co, Cu, Fe, Mg, Ni and Pb, t-test, *p* < 0.05) (Fig. 2). Moreover, mosses from this category differed significantly from those collected from the control site in terms of the concentration if all elements except K and Mn (t-test, *p* < 0.05) indicating that irrigated transplants of *H. splendens* are efficient trace element accumulators after 3 months of exposure. To distinguish levels of the accumulation of elements by mosses in three categories of research sites relative accumulation factors (RAF) were calculated (Table 1), which helped to avoid the influence of the initial element content (Aničić at al. 2009). For industrial sites RAF factors were arranged in the order: Pb (highest) > Cd > Fe > Zn > Co > Mg > Ni > Cu > V > Cr > K > Mn for industrial, Pb > Cd > Zn > Fe > Co > Cr > Mg > Ni > V > Cu > K > Mn for urban and Pb > Cd > Zn > Fe > Mg > Co > Cr > Cu > K > Ni > V > Mn for rural sites. The highest RAF values were obtained for industrial sites for most of the elements, but the order of the elements was very similar for all the categories of research sites, which shows that the *H. splendens* mosses accumulated mainly Pb, Cd, Zn and Fe (highest RAF). It was determined that Cd, Pb and Zn showed high covalent indexes (Boquete et al. 2015) and formed strong bonds with cation exchange sites in moss, so that the elements would not be easily displaced by other trace elements, leading to higher bioconcentration. It can be concluded that the elevated concentrations of the studied elements were transported with air and they could affect the chemistry of mosses collected from all study sites. In addition, most likely the emission of these elements came from the same origin. These relative accumulation factors, found especially at industrial sites, reflected the emissions produced by the heavy industry of Upper Silesia. Pollution caused by Pb, Cd and Zn is characteristic especially of the ore mining area around Olkusz in Southern Poland (site 11, Fig. 1). This area was contaminated by the exploitation and processing (mining and smelting) of these ores (Cabala et al. 2009). Part of Zn in mosses may probably be attributed also to contribution from higher vegetation (Berg et al. 1995). Zinc ores and fossil fuels (e.g. hard coal) contain a significant amount of Cd. Moreover as a result of their extraction and processing, significant amounts of Cd are released into the atmosphere, hydrosphere and soils. In the case of metallurgical industry, also present in the studied area, waste gases withdrawn from the sinter strand during production processes carry dusts containing heavy metals (mainly Fe, Cd, Zn, Pb, Ni and Cr). Therefore it is not surprising that the RAF values determined for industrial category sites were higher (especially for Pb, Cd, Zn and Fe) than in the majority of other studies (Hu et al. 2018). In moss transplants collected from all categories of sites loss of Mn due to washing out and leaching (Aničić et al. 2009) with respect to the initial material was evident. Some researchers in previous studies attempted to calibrate moss concentrations against bulk deposition to obtain absolute deposition values (e.g. Berg et al. 1995). Berg and Steinnes (1997) indicate that atmospheric humidity and precipitation play an important role as factors for moss accumulation. In this research significant correlations between element concentrations in mosses and bulk deposition were found for Cd, Ni, Pb and Zn (industrial sites), Cr, Fe and Zn (urban sites), Fe and Pb (rural sites). Aboal et al. (2010) found high correlations for Cd, Pb and Zn in native mosses. Berg et al. (1995) and Berg and Steinnes (1997) also reported significant correlations between wet deposition of Fe, Cd and Pb and their concentrations in the same moss species (*H. splendens*). However in the study of Berg et al. (1995), the authors concluded that no significant correlations between mosses and wet deposition were seen for Cr, Fe and Ni, which may be due to the dominant sources other than atmospheric precipitation for these elements in *H. splendens* growing in Norwegian background stations. On the contrary, in this study relationships for these elements were found, which may indicate a significant impact of precipitation on the chemistry of mosses in Upper Silesia. In the study of Aničić et al. (2009) carried out in urban areas of Belgrade (Serbia) Cr, Fe and Zn in mosses were correlated with the content of these elements in precipitation. Despite the fact that moss analysis normally does not enable the estimation of absolute trace element deposition rates for most elements in the way precipitation analysis does (Berg and Steinnes 1997), it can serve as a valuable source of information about changes in environmental chemistry. Poor or no correlations of moss vs bulk deposition for some elements can be attributed to more complex processes of element accumulation in mosses due to their morphological and physiological characteristics (Aničić et al. 2009). The analysis of precipitation samples provides data on total trace elements deposited, and the analysis of mosses shows the concentration of trace elements accumulated and retained by mosses (Boquete et al. 2015). Therefore, moss analysis might be more accurate in describing qualitative information about the studied areas. Trace element concentrations in mosses are not only explained by concentrations in bulk deposition, but elements could be deposited onto moss surface also as dry particulates and retained by particulate entrapment. It is worth to mention that *H. splendens*, which has small leaves and is highly branched, is regarded as an excellent trap for ‘minerogenic’ elements among moss species (Halleraker et al. 1998). Several studies (e.g. Steinnes 1995) mentioned the importance of local dust for the observed moss chemistry. The concentration of elements in dust samples (Table 2) does not show any variability in the duration of the whole study (t-test; *p* < 0.05). The number of significant correlations between mosses and dust deposition was higher than for bulk deposition (mosses vs. dust deposition: Cd, Cr, Cu, Mn, Ni, Pb and Zn in industrial sites; Cu, Fe, Mn, Ni, Pb and Zn in urban sites, Cd, Cr, Cu, Pb and Zn in rural sites). Theodosi et al. (2010) determined the proportion of elements between wet and bulk deposition and concluded that Cr and Pb were mainly associated with the particulate form (64%–98%). In this study dry deposition carried higher element concentrations than wet deposition, which could result in the higher passive capture of particulate matter and entrapment of elements by mosses. Dry deposition is an important source of elements for mosses, and it was shown that they may intercept up to 98% of PM10 particles (Tretiach et al. 2011). It is suggested that particles trapped by mosses may be the major source of poorly water-soluble elements and that moss content can reflect recent environmental conditions for dry and coarse depositions, especially for active biomonitoring studies in highly polluted areas (Aničić et al. 2009). Although the concentration of elements in bulk and dust deposition is affected by environmental conditions, comprehensive assessment of atmospheric quality is only possible after the results are supplemented with the airborne dust bulk precipitation level. Research results show that the quality of the air in industrial, urban and rural areas is determined first of all by the influence of local sources.
